# The Prediction and Validation of Small CDSs Expand the Gene Repertoire of the Smallest Known Eukaryotic Genomes

**DOI:** 10.1371/journal.pone.0139075

**Published:** 2015-09-30

**Authors:** Abdel Belkorchia, Cyrielle Gasc, Valérie Polonais, Nicolas Parisot, Nicolas Gallois, Céline Ribière, Emmanuelle Lerat, Christine Gaspin, Jean-François Pombert, Pierre Peyret, Eric Peyretaillade

**Affiliations:** 1 Clermont Université, Université d'Auvergne, Laboratoire "Microorganismes: Génome et Environnement", BP 10448, F-63000, Clermont-Ferrand, France; 2 CNRS, UMR 6023, LMGE, F-63171, Aubière, France; 3 Clermont Université, Université d’Auvergne, EA 4678 CIDAM, BP 10448, F-63001, Clermont-Ferrand, France; 4 Biologie Fonctionnelle Insectes et Interactions, UMR203 BF2I, INRA, INSA-Lyon, Université de Lyon, Villeurbanne, France; 5 Université de Lyon, F-69000, Lyon, Université Lyon 1, CNRS, UMR 5558, Laboratoire de Biométrie et Biologie Évolutive, F-69622, Villeurbanne, France; 6 INRA, UBIA UR 875, F-31320, Castanet-Tolosan, France; 7 Illinois Institute of Technology, Department of Biology, 3105 South Dearborn Street, Chicago, Illinois, 60616, United States of America; University of North Carolina at Charlotte, UNITED STATES

## Abstract

The proper prediction of the gene catalogue of an organism is essential to obtain a representative snapshot of its overall lifestyle, especially when it is not amenable to culturing. Microsporidia are obligate intracellular, sometimes hard to culture, eukaryotic parasites known to infect members of every animal phylum. To date, sequencing and annotation of microsporidian genomes have revealed a poor gene complement with highly reduced gene sizes. In the present paper, we investigated whether such gene sizes may have induced biases for the methodologies used for genome annotation, with an emphasis on small coding sequence (CDS) gene prediction. Using better delineated intergenic regions from four *Encephalitozoon* genomes, we predicted *de novo* new small CDSs with sizes ranging from 78 to 255 bp (median 168) and corroborated these predictions by RACE-PCR experiments in *Encephalitozoon cuniculi*. Most of the newly found genes are present in other distantly related microsporidian species, suggesting their biological relevance. The present study provides a better framework for annotating microsporidian genomes and to train and evaluate new computational methods dedicated at detecting ultra-small genes in various organisms.

## Introduction

The accurate prediction of genes is a fundamental step in the determination of all biological processes that govern organism life [1]. Unfortunately, small protein-coding genes are often overlooked by annotation projects in an effort to minimize over-predictions due to their shortness, dearth of primary sequence conservation and/or lack of known functions [2–4]. Major algorithms to annotate, organize and functionally characterize such genes have been described recently [5, 6], but the *in silico* determination of small CDSs (Coding DNA Sequences; sCDSs ≤ 300 nucleotides) remains challenging. Nevertheless, the biological relevance of sCDSs should not be understated. Proteins translated from sCDSs were found to have a much richer functional spectrum than anticipated in both prokaryotes and eukaryotes [7–9] and, for example, effector genes in fungi, oomycetes and bacterial pathogens code for products involved in subverting the host cell biology during infection, and so play a tremendous role in pathogenicity [10, 11].

Microsporidia are ubiquitous, eukaryotic and opportunistic intracellular parasites [12] clustering at the base of the fungal kingdom as a sister-group to chytrid pathogen *Rozella allomycis* [13]. The microsporidian phylum includes over 1500 species of medical, veterinary and economic impacts that infect all animals and which induce various systemic diseases in the afflicted hosts [14]. In general, microsporidian genomes are gene poor [15–28] and these obligate parasites must rely on their host for a number of essential cellular components that they are no longer able to produce [12, 29, 30]. However, despite drastic gene losses in all members of the phylum, their genome sizes vary extensively, from 24 Mbp in *Hamiltosporidium* spp. to less than 3 Mbp for species belonging to the genus *Encephalitozoon*. This variation in size has been attributed mostly to genome duplications [12, 27, 29, 30], to the acquisition of new genes by horizontal transfer from different prokaryotic and eukaryotic donors [23, 28, 31–33], to expansions/contractions in intergenic regions [15, 34], and to the propagation of transposable elements [24, 27, 33].

Until now, microsporidian genomes have been annotated using *ab initio* protein predictions that were based primarily on the detection of open reading frames (ORF) displaying homology with coding regions of functional importance or, alternatively, of a minimum target length. Highly divergent orthologs between these organisms were also inferred based on gene order conservation [35, 36] and a recent study using transcriptional signals coupled to comparative genomic analyses highlighted 110 additional genes (around 5.5%) in the microsporidian species *Encephalitozoon cuniculi* [24].

Here, we investigated the presence of unannotated sCDSs genes in Microsporidia using as reference models the publicly available genomes from four distinct species belonging to the genus *Encephalitozoon*. These genomes were chosen because of their evolutionary and medical importance and extremely compact state. Indeed, the *Encephalitozoon* genomes are both very small (2.3–2.9 Mbp) and compact (120 bp intergenic spacers on average), and encode fewer proteins than their eukaryote counterparts (~ 1900 CDSs). Their genomes are also highly syntenic with large blocks of genes arrayed identically, and we hypothesized that the conserved sCDSs located therein would be easier to distinguish from regions with lower functional constraints (*e*.*g*. non-coding regions) due to the high rate of sequence evolution that occurs between the four *Encephalitozoon* species [15, 19, 23, 24]. Specifically, the *Encephalitozoon* intergenic regions were precisely delimited using refined CDS annotations based on transcriptional signals [24, 37] and then compared to highlight the presence of elevated sequence conservation likely to indicate functional importance. Putative novel sCDSs thus inferred were confirmed by RACE-PCR transcript characterization in *Encephalitozoon cuniculi*. This study underlines the usefulness of sequencing closely related species (e.g. within the same genus) to help identify small but probably essential genes.

## Materials and Methods

### Cell culture and RNA extraction

Confluent Human Foreskin Fibroblast (HFF) host cells (ATCC SCRC-1041)were infected by approximately 10^9^ spores of *E*. *cuniculi* GB-M1 (kindly provided by Prof. Elisabeth U. Canning, Imperial College of Science, Technology and Medicine, London, UK) during 2 hours in 75 cm^2^ flasks. Cultures were washed three times with PBS (1X) to eliminate spores that did not invade host cells and incubated for 2 days as described previously [38]. Infected cells were then maintained in 5% CO_2_ at 37°C in minimum essential medium (MEM) supplemented with 5% foetal calf serum, 2 mM glutamine (Invitrogen, Carlsbad, CA, USA) and 20 μg/ml gentamicin. Total RNA was extracted using RNeasy Midi Kit (Qiagen, Venlo, Limburg, Netherlands) as described previously [37].

### RACE-PCR experiments

Putative mRNA ends were amplified by 5' and 3’ RACE PCR with the SMARTer RACE Amplification kit (Clontech Laboratories, Inc., Mountain View, CA, USA) according to the manufacturer recommendations. RT reactions steps were performed with 500 ng of *E*. *cuniculi* total RNA extracted from infected cells using the modified oligo-d(T) primers provided by the SMARTer RACE Amplification kit. First strand reaction products were diluted with 50 μl of tricine-EDTA buffer. These RT products were then used for PCR amplifications with specific gene primers (0.2 μM, 0.2mM dNTPs, 2 U Taq polymerase) on an Eppendorf Mastercycler gradient PCR machine with the following cycling parameters: 10 cycles of touch-down PCR (denaturation: 94°C for 30s; annealing: 68–55°C for 30s; extension: 72°C for 30s), followed by 30 cycles of regular PCR with annealing at 52°C. Specific gene primers were defined using the KASpOD software [39].

### PCR products sequencing

Presence and size of the amplification products were determined by electrophoresis on 1.5% agarose gels. Bands of the expected sizes were excised and purified using the Wizard SV Gel and PCR Clean-Up System (Promega, Madison, WI, USA). Purified PCR products were directly sequenced with the specific primers from the RACE amplifications. In some case, PCR products were ligated into the pCR II TOPO vector (TOPO TA Cloning Kit Dual Promoter, Invitrogen) and transformed into chemically competent XL1-Blue *Escherichia coli* cells following the Inoue method [40]. All sequences were determined using the Sanger dideoxynucleotides chemistry by MWG Operon (Ebersberg, Germany) with the SP6 primers.

### Sequence analyses

To accurately identify and delineate coding and intergenic regions, each predicted protein sequence from the four *Encephalitozoon* genomes was used as query for BLASTP and TBLASTN analyses [41] against the three remaining proteomes and genomes, respectively. Protein and nucleotide alignments between orthologs were performed with MUSCLE 3.8.31 [42] and Clustal Omega [43] respectively. Their proper start/stop codons were then curated manually using the Artemis [44] annotation platform. Intergenic regions were extracted from the curated annotations using the custom Perl script and module intergenic_extract.pl and CDS.pm, respectively (https://github.com/EACIDAM/perl_script/blob/master/). Small proteins were detected using an “all-*versus*-all” TBLASTX approach (BLOSUM45, word size: 2 aa, low-complexity filter disabled) whereas putative transcriptional signals were manually searched for in the upstream and downstream regions of each predicted CDS. Multiple sequence alignments between newly predicted orthologs from these four genomes were performed with MUSCLE 3.8.31. Orthologous proteins from other species were retrieved by a PSI-BLAST approach (three iterations, BLOSUM45) [45] using custom microsporidian and fungal databases. The microsporidian database was built from the genome sequences of 13 species extracted from the NCBI database: *Nematocida parisii*, *Anncaliia algerae*, *Nosema bombycis*, *Hamiltosporidium tvaerminnensis*, *Ordospora colligata*, *Vittaforma corneae*, *Nosema apis*, *Spraguea lophii*, *Edhazardia aedis*, *Vavraia culicis*, *Nosema ceranae*, *Mitosporidium daphniae*, and *Trachipleistophora hominis*. The fungal database was created from the NCBI RefSeq release version 01/2015 (ftp://ftp.ncbi.nlm.nih.gov/refseq/release/fungi/). ORFs of at least 69 nt were extracted with Getorf from the EMBOSS 6.6.0.0 package [46]. Conserved domains in the small protein-coding genes were predicted using InterProScan 5 [47], Pfam 27.0 [48], SignalP 4.1 [49] and TMHMM2.0 [50]. Maximum likelihood phylogenetic inferences based on the gene coding for the small ribosomal RNA subunit were performed under the HKY85 model of nucleotide substitution as implemented in PhyML 3.0 [51]. For this analysis, the orthologous sequences were first aligned with MAFFT version 7 [52] and the ambiguous regions in the alignments were filtered out with TrimAL version 1.3 using the automated1 parameter [53].

## Results

To facilitate the detection of small functional open reading frames, previously overlooked in Microsporidia we first performed a thorough curation of the available *Encephalitozoon* genome annotations. Using data from the four available *Encephalitozoon* genomes [15, 19, 23, 24], a total of 2, 2, 82, and 75 CDSs were added to the *E*. *cuniculi*, *E*. *intestinalis*, *E hellem* and *E*. *romaleae* annotations, respectively (S1 Table). Using these comparative extrinsic data, we also identified 57, 51, 44 and 139 translation initiation sites (TISs) in *E*. *cuniculi*, *E*. *intestinalis*, *E*. *hellem* and *E*. *romaleae*, respectively (S2 Table).

**Fig 1:**
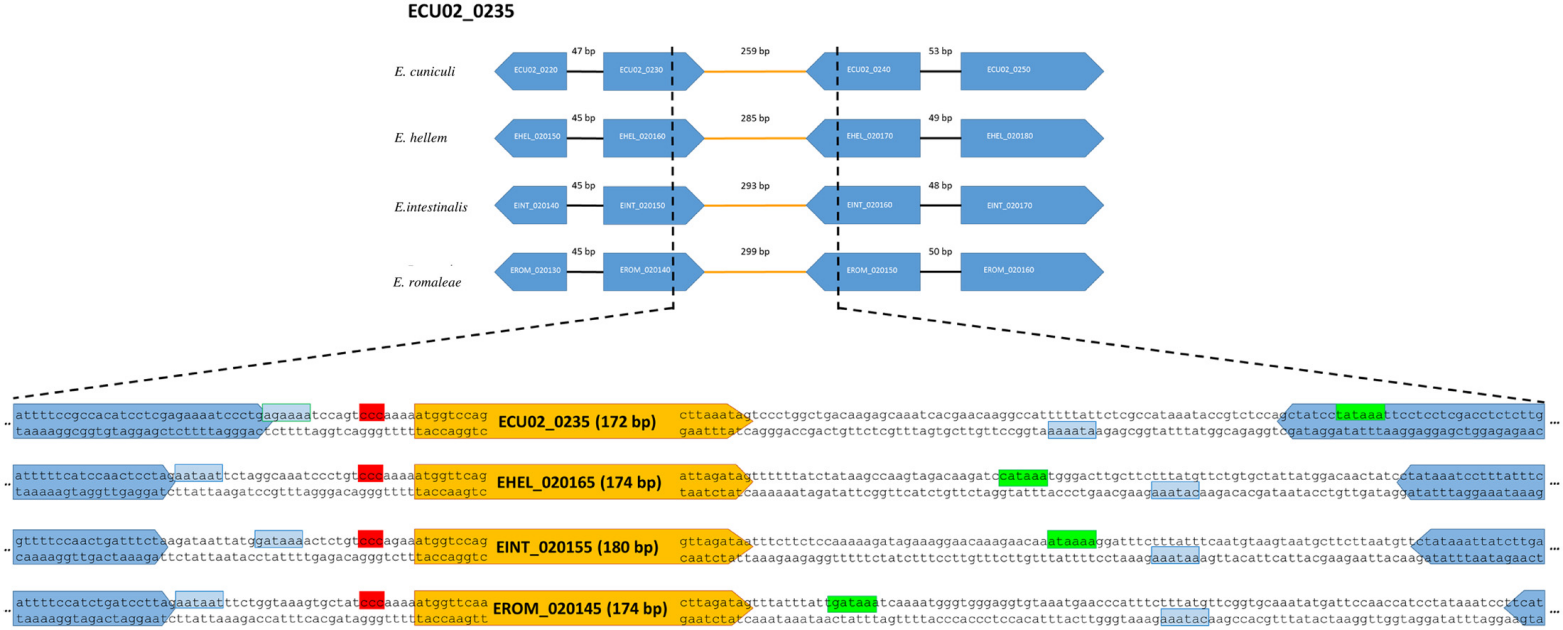
Example of the genomic context of previously annotated genes and newly-identified sCDSs in *Encephalitozoon* genomes. The transcriptional signals of the newly predicted genes are highlighted in red (promoter signal) and green (polyadenylation signal), respectively. The putative polyadenylation signals of the genes flanking the new sCDSs are highlighted in light blue.

**Fig 2:**
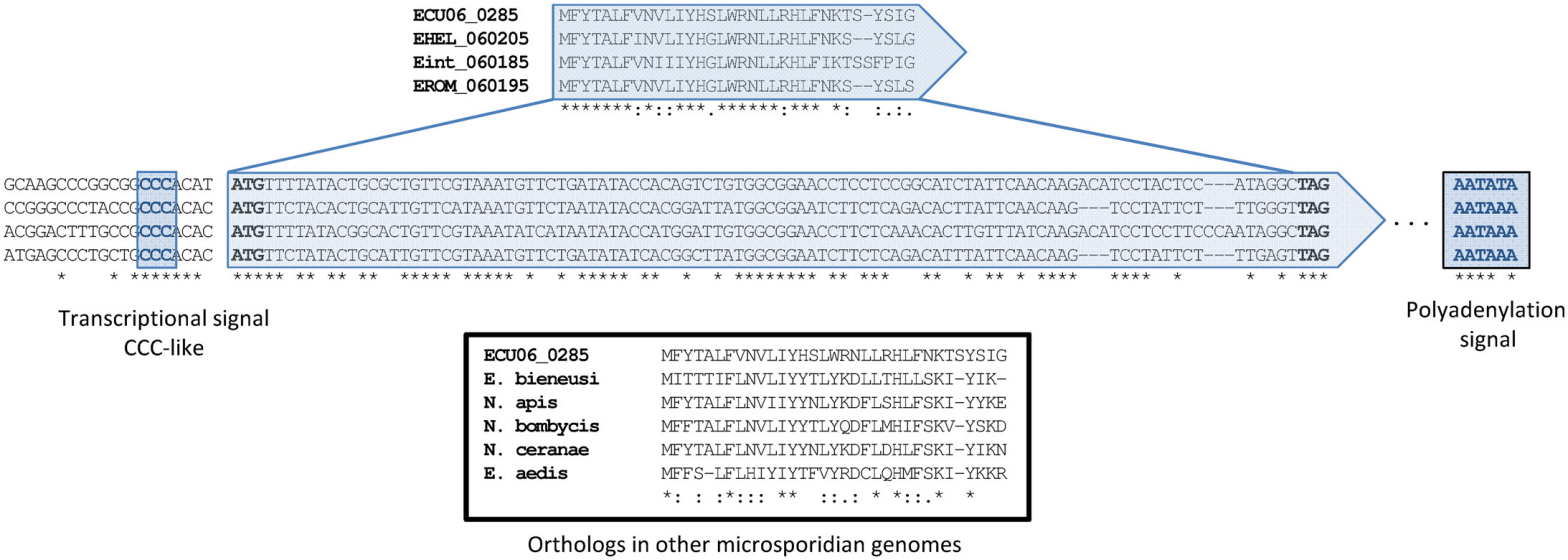
Validation example of the newly predicted orthologs using both protein and nucleotide sequence alignments. Protein and nucleotide alignments were performed using MUSCLE and Clustal Omega, respectively.

Thereafter, using the curated annotations described above, we searched for the presence of short protein-coding gene candidates. Specifically, we searched for transcriptional and/or translational signals in intergenic regions that flanked small open reading frames, with the condition that both signals and ORFs were conserved across the *Encephalitozoon* genomes. Using this approach, a total of 31 small but highly conserved CDSs were identified in the four *Encephalitozoon* species (Fig 1, Table 1 and S3 Table). Another sCDS was also found to be shared between *E*. *cuniculi* (ECU04_1635) and *E*. *romaleae* (EROM_041665). However, its presence could not be ascertained in *E*. *hellem* and *E*. *intestinalis* because its location, based on syntenic information, falls within unsequenced regions. The proteins encoded by the newly-identified small CDS range from 25 to 84 amino acids in *E*. *cuniculi* (median 55; Table 1) and generally show a high level of similarity across the four *Encephalitozoon* species, with an average of 72% (min 46%, max 96%; Fig 2 and S1 Fig).

**Table 1:** Predicted small protein-coding gene orthologs in the four *Encephalitozoon* species. Orthologs in other microsporidian genomes were predicted using PSI-BLAST and manual validation. Additional functional inferences were performed using InterProScan 5 (conserved amino-acids motifs), TMHMM (transmembrane helices) and SignalP (signal peptides). Bold: Genes present in independent RNA-Seq datasets [48].

Locus tag				Gene product size (aa)	Microsporidian species with orthologs ^(1)^	AAATTT or Adenine/Thymine rich signals for *E*.*cuniculi*	Interpro domain	TMHMM	SignalP
*E*. *cuniculi*	*E*. *intestinalis*	*E*. *hellem*	*E*. *romaleae*						
ECU01_1065	Eint_010975	EHEL_010945	EROM_010865	73	Th^a^, Vc, Aa,Oc				
ECU02_0235	Eint_020155	EHEL_020165	EROM_020145	57	Ea, Na, Nb, Nc, Th^a^, Vco, Aa, Ht, Vc, Oc				
ECU02_0425	Eint_020355	EHEL_020345	EROM_020335	57				+ (1)	
ECU02_0885	Eint_020835	EHEL_020805	EROM_020795	59	Oc			+ (1)	
**ECU02_1495**	**Eint_021465**	**EHEL_021435**	**EROM_021405**	**56**	**Ea, Na, Nb, Th** ^**a**^ **, Vc, Ht, Nc, Vco, Oc, Sl**	**+**			
**ECU03_0255**	**Eint_030145**	**EHEL_030135**	**EROM_030155**	**56**	**Oc**		**IPR013829**		
ECU04_0123	Eint_040045	EHEL_040035	EROM_040065	55	Oc			+ (1)	
ECU04_0152	Eint_040082	EHEL_040072	EROM_040102	54	Th, Vc, Np				
ECU04_1622	Eint_041635	EHEL_041595	EROM_041652	28	Na, Nb, Aa, Ea, Nc, Th, Vc, Oc, Vco				
ECU04_1635	—	—	EROM_041665	55					
**ECU05_0087**	**Eint_050075**	**EHEL_050137**	**EROM_050055**	**71**	**Aa, Vc, Ea, Sl, Th, Oc, Na, Nb** ^**a**^ **, Ht**	**+**			**+**
ECU05_0115	Eint_050105	EHEL_050165	EROM_050085	65					
ECU05_1185	Eint_051235	EHEL_051295	EROM_051225	51	Nc, Ea, Na, Nb, Oc				
ECU05_1275	Eint_051335	EHEL_051395	EROM_051335	42	Oc				
ECU06_0285	Eint_060185	EHEL_060205	EROM_060195	33	Eb, Na, Nb, Nc, Ea			+ (1)	
ECU07_0862	Eint_070802	EHEL_070832	EROM_070812	41					
ECU07_1385	Eint_071345	EHEL_071365	EROM_071325	84	Aa, Ea, Eb^a^, Na^a^, Nb^a^, Nc, Np^a^, Sl^a^, Th, Vc, Vco, Ht^a^, Oc		IPR024766		
ECU07_1645	Eint_071493	EHEL_071625	EROM_071565	69					
ECU07_1775	Eint_071493	EHEL_071755	EROM_071695	75		+		+ (1)	
**ECU08_1445**	**Eint_071493**	**EHEL_081425**	**EROM_081445**	**60**	**Oc, Nc, Nb** ^a^ **, Na**				
**ECU08_1555**	**Eint_071493**	**EHEL_081525**	**EROM_081555**	**52**	**Aa, Ea** ^a^ **, Eb** ^a^ **, Ht, Na, Nb** ^a^ **, Nc, Np** ^a^ **, Sl** ^a^ **, Th, Vc** ^a^ **, Vco** ^a^ **, Oc**		**IPR007264**		
ECU09_0465	Eint_090475	EHEL_090465	EROM_090475	42					
**ECU09_1255**	**Eint_091465**	**EHEL_091435**	**EROM_090625**	**25**					
ECU09_1665	Eint_091675	EHEL_091675	EROM_091655	49	Aa, Sl, Th, Vc, Oc				
ECU09_1755	Eint_091775	EHEL_091775	EROM_091755	43	Nc, Oc, Na, Ea				
**ECU10_0635**	**Eint_100575**	**EHEL_100635**	**EROM_100505**	**68**	**Oc**				
ECU11_0185	Eint_110055	EHEL_110065	EROM_110055	42	Nb, Nc, Eb^a^, Na, Vco, Oc				
ECU11_0525	Eint_110375	EHEL_110395	EROM_110385	25	**Nb** ^a^ **, Oc**				
ECU11_0575	Eint_110425	EHEL_110445	EROM_110435	49	Oc				
ECU11_1175	Eint_111055	EHEL_111055	EROM_111055	84	Oc				
**ECU11_1205**	**Eint_111085**	**EHEL_111085**	**EROM_111085**	**43**	**Vco, Th** ^a^ **, Nb, Vc, Ea, Ht, Sl, Aa, Na, Oc**				
**ECU11_1725**	**Eint_111615**	**EHEL_111615**	**EROM_111615**	**68**	**Th, Vc**	**+**			

^a^ Previously predicted

^(1)^ Accession numbers, positions and locus tags are listed in the S3 Table.

Aa (*Anncaliia algerae*); Ea (*Edhazardia aedis)*; Eb (*Enterocytozoon bieneusi*); Ht (*Hamiltosporidium tvaerminnensis*); Oc (*Ordospora colligata*); Na (*Nosema apis*); Nb (*Nosema bombycis*), Nc (*Nosema ceranae*); Np (*Nematocida parisii*); Sl (*Spraguea lophii*); Th (*Trachipleistophora hominis*); Vc (*Vavraia culicis*); Vco (*Vittaforma cornae*)

**Fig 3:**
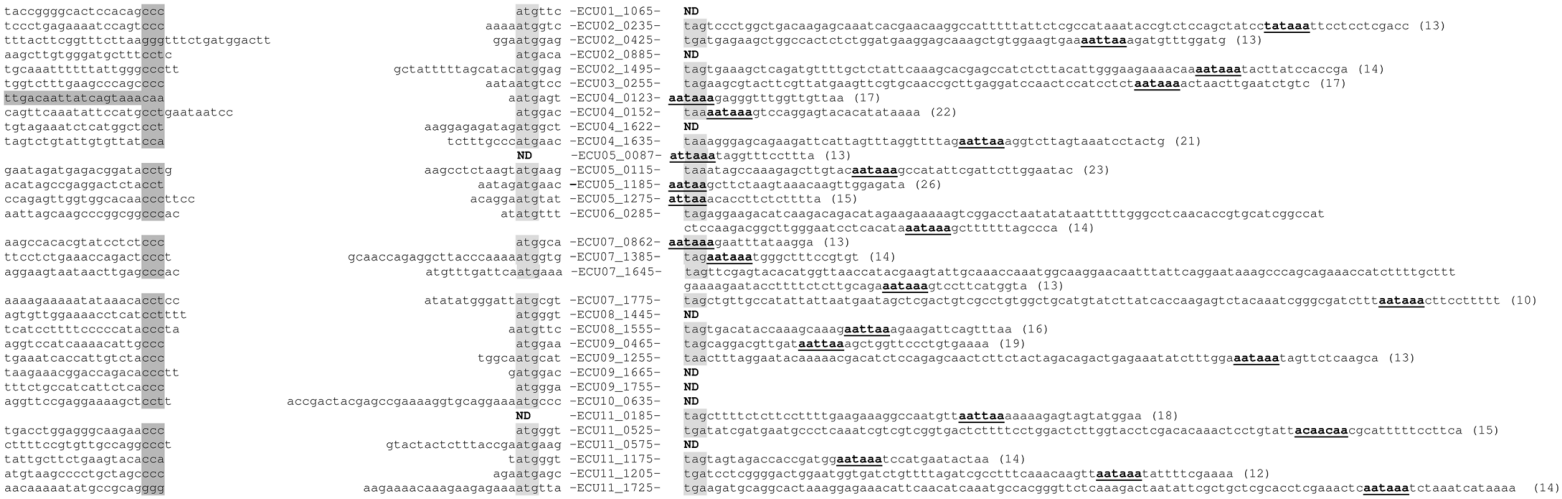
Identification of the 5' and 3' maturation sites of the newly predicted small CDSs. Translation initiation codons and stop codons are highlighted in light-grey for all genes. Putative polyadenylation signals are underlined and highlighted in bold characters. Distances between putative polyadenylation signals and polyadenylation sites are indicated between parentheses. Putative microsporidian promoter specific signals, located upstream the transcription start sites, are highlighted in dark grey. For brevity, the complete CDS sequences were not included and are represented instead by the corresponding gene names. ND; Not Defined.

All of the 32 predicted sCDSs were confirmed to be transcribed in *E*. *cuniculi* by 5’ and/or 3’ RACE-PCR experiments followed by Sanger sequencing of the RACE-PCR products thus obtained (Fig 3). The 5’ transcriptional start and polyadenylation sites have been identified for most genes, including those (ECU02_0425, ECU04_1635, ECU05_0115, ECU07_0862, ECU07_1645, ECU07_1775, ECU09_0465 and ECU09_1255) for which no ortholog could be detected in other non-*Encephalitozoon* microsporidian species (Fig 2 and Table 1). A total of four genes (ECU02_1495, ECU05_0087, ECU07_1775 and ECU11_1725) were also found to harbor upstream of their CCC-like motif, adenine/thymine-rich AAATTT-like or adenine rich sequences that are positively correlated with high gene expression levels in Microsporidia [37]. Thus, integrating all of these results we propose that *E*. *cuniculi*, *E*. *intestinalis*, *E*. *romaleae* and E. *hellem* contain 2126, 1927, 1904 and 1955 CDSs, respectively.

**Fig 4:**
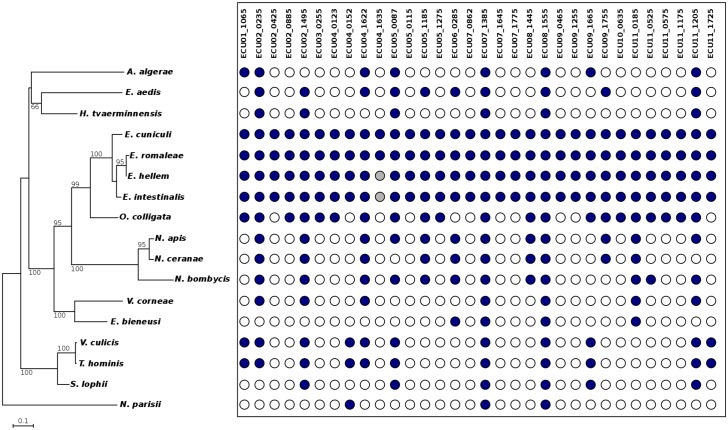
Phylogenetic distribution of the newly predicted small protein-coding genes across 17 sequenced microsporidian species. *Left*: The HKY85 Maximum Likelihood phylogenetic tree shown here is derived from the small ribosomal RNA-encoding gene. Bootstrap support for each cluster is indicated on the corresponding nodes; only bootstraps greater than 50% are indicated. *Right*: The presence/absence of the newly identified sCDSs in the corresponding species are denoted by filled and empty circles, respectively. The two grey circles indicate genes that fall within unsequenced regions in the *E*. *intestinalis* and *E*. *hellem* genomes and whose presence could not be confirmed. Locus names of the new sCDSs (on top) are derived from the *E*. *cuniculi* accessions.

Despite the high rate of sequence evolution prevalent in Microsporidia, we were able to discern putative homologues in non-*Encephalitozoon* microsporidian species for 24 of the 32 newly identified sCDSs (Fig 4; Table 1; S3 Table). Putative orthologs of ECU02_1495, ECU07_1385, ECU08_1555 and ECU11_1205 were also found in non-microsporidian fungi. A hemaglutinin glycoprotein domain (IPR013829; ECU03_0255) potentially involved in pathogenicity and host invasion, a Zinc finger domain (IPR024766; ECU07_1385) involved in protein-protein or protein-DNA interactions and a nucleolar protein NOP10-like domain (IPR007264; ECU08_1555) involved in 18S rRNA production or rRNA pseudo-uridylation were found in the predicted proteins. Although no protein domain was detected in the ECU02_1495 microsporidian sequence, the similarly-sized putative homologs identified in other fungi harbor the Mozart1 Pfam domain (PF12554). This protein family operates as part of the gamma-TuRC gamma-tubulin ring complex composed of six subunits and which is involved in chromosome segregation during mitosis [54]. Single transmembrane domains were also identified in five proteins (ECU02_0425, ECU02_0885, ECU04_0123, ECU06_0285 and ECU07_1775; Table 1). Only one protein, ECU05_0087, was predicted to display a signal peptide.

## Discussion

Size and perspective are two important factors defining the space of a search. Needles are intuitively easier to find in their sleeves than in a haystack, and horses are easier to find in haystacks than needles. But when we do not know what we are searching for looks like, the difficulty of the search is compounded. Large, highly conserved genes are a lot easier to find than small and derived ones, and the larger the genome the more difficult this process becomes. Small protein-coding genes are often overlooked for their size renders them hard to distinguish from spurious hits, especially when they lack known functions. Dedicated algorithms for identifying small genes with high coding potential currently suffer from a high false positive rate [4] and both experimental and computational studies are required to further advance their accuracy. Considering that small genes account for over 5% of the *Saccharomyces cerevisiae* genome coding capacity [45], they are far from irrelevant. In this study, we used the availability of multiple closely related *Encephalitozoon* genomes as well as the presence of transcriptional signals to improve gene prediction from intergenic regions.

The *in silico* approach we used here proved particularly successful at avoiding false positives, as all of the predicted proteins were confirmed by RACE-PCR and sequencing. The overall number of new genes that we identified here may appear small when compared to the 1900+ proteins encoded by these genomes, but this number is higher than we expected at the start of this study. The *Encephalitozoon* genomes are models of compactness that have been studied extensively over the years, such that the total number of genes we found exceeded our expectations. These results also highlight the relevance of revisiting genome annotations periodically as additional genomes are being released to improve existing annotations by comparative approaches. The approach we used here should be amenable to most microsporidian genomes as their transcriptional and translational processes are controlled by conserved regulatory elements [24, 37].

Transcriptomics approaches are routinely used to assist genomic annotations of higher eukaryotes in order to find and precisely delimit introns and exons junctions. Those approaches however, are less commonly used with microsporidia due in large part to the paucity of introns they harbor and to the difficulty of isolating the meronts from their hosts. Nevertheless, 9 of the 32 small genes that our approaches have identified here were also found present, in independent *E*. *cuniculi* RNA-Seq experiments [55], thus providing external confirmation that these were not procedural artefacts. While the remaining 23 genes were not found in this external dataset, these may simply correspond to genes that are either lowly expressed or expressed under conditions that differ from the performed RNA-Seq experiments, in which the RNA was isolated at three specific post-infection time points [55]. Another possibility is that these transcripts were present but discarded by the pipelines used due to the filtering schemes involved (e.g. the removal of transcripts shorter than a specified cutoff).

Some of the sCDS identified here in this manuscript were accurately predicted in the *N*. *bombycis* and *T*. *hominis* genomes with *ab initio* gene prediction methods [22, 27]. However those methods also likely lead to numerous false positive predictions, for the large number of genes in *N*. *bombycis* and *T*. *hominis* coupled with their unusually small average sizes suggest an over-prediction of small genes. In *Encephalitozoon* species, the mean CDS length for the 2000 or so proteins is close to 1000 bp ([24, 34] and this study) but in *N*. *bombycis* and *T*. *hominis*, the mean CDS lengths are noticeably lower, at 741 and 871 bp, respectively [22, 27]. Out of the 4,458 and 3,266 predicted proteins in *N*. *bombycis* and *T*. *hominis*, 718 and 736 code for proteins that are smaller than 100 amino acids. Of these, less than 30% displayed any homology to conserved domains or known proteins, suggesting that the default trade-off between specificity and sensitivity of the corresponding *ab initio* prediction software was suboptimal for Microsporidia.

Unfortunately, we couldn’t assign functions to many of the newly found CDS. Functional inferences based on homology are only as good as their reference datasets, and while small CDSs have been identified in animals, plants, yeasts, and bacteria, their functions have been rarely addressed [56]. Microsporidia currently lack a viable genetic characterization system, unlike many model organisms, and the RNA interference machinery is absent from *Encephalitozoon* species, preventing identification through silencing. Other Argonaute/PIWI-bearing microsporidian species do exists, but RNA interference assays have yet to be implemented in Microsporidia. That said, the presence of a hemaglutinin glycoprotein potentially involved in pathogenesis among the putative functions that we were able to infer suggests that exploring these sCDS further will likely yield profitable insights into the parasitic cycle of these organisms. At the very least, localization experiments using antibodies should give us a glimpse into their biological functions.

## Conclusion

This study underlines the usefulness of associating classic gene prediction and fine genome exploration (e.g. synteny, transcriptional signals) to improve annotation in Microsporidia. Recently, a similar approach has been successfully used to perform identification of sCDSs from the re-sequencing of eight isolates of *N*. *ceranae* species [57]. Both independent studies highlight the value of sequencing very phylogenetically closely-related species to reveal their complete gene repertoires, an essential step towards the understanding of an organism physiology and adaptive capabilities. This is especially true when the species involved are fast evolving organisms and/or hard to culture such as microsporidia. Finally, the current study provides an important framework for future studies and datasets that can be used to better train and evaluate new computational methods dedicated at detecting ultra-small genes.

## Supporting Information

S1 Fig *Encephalitozoon* protein sequences and multiple alignments (obtained with MUSCLE). (DOCX)

S1 Table List of newly predicted protein genes in the *E*. *cuniculi*, *E*. *intestinalis*, *E*. *hellem* and *E*. *romaleae* genomes based on extrinsic data. (XLSX)

S2 Table List of genes with readjusted TIS in the *E*. *cuniculi*, *E*. *intestinalis*, *E*. *hellem* and *E*. *romaleae* genomes. (XLSX)

S3 Table Genes newly identified in the *Encephalitozoon* genus and their orthologs in other microsporidian genomes. (XLSX)
