# Supplementary material for: The Prediction and Validation of Small CDSs Expand the Gene Repertoire of the Smallest Known Eukaryotic Genomes
Source: PLoS One. 2015 Sep 30;10(9):e0139075. doi: 10.1371/journal.pone.0139075 (PMC4589312; doi:10.1371/journal.pone.0139075)
Supplement: S1 Fig — (DOCX) [file pone.0139075.s001.docx]

**Supplementary Fig S1. Protein sequences and alignments in *Encephalitozoon* genus obtained by MUSCLE.**

**Chromosome 1**

| Species | Positions |
| --- | --- |
| *E. cuniculi* | 123947..124168 |
| *E. hellem* | 98768..98950 |
| *E. intestinalis* | 99143..99334 |
| *E. romalae* | 85793..85975 |

>ECU01_1065

MFNMDHTYYTDAFLDACLLGHTTIELTAKPFTNIKDEDIEEAPIPLARDQAFPIDFGLTP

WERQDQASKRINN

>EHEL_010945

MFTMDHTYYTDAFLDACLLGYTSIELTASSFTNIKDEEIEEVPIPLAKDHAFPIDFGLGP

>EINT_010975

MFVQDHTYYTDAFLDACLLNFERIELTAKPFTNIKDEDLEESPVPLNKDHPFPIDFELTP

RNA

>EROM_010865

MFEMDHTYYTDAFLDACLLGYTSIELTAKPFTNVKDEDIEETPIPLTKDHVFPIDFGLTL

EINT_010975 MFVQDHTYYTDAFLDACLLNFERIELTAKPFTNIKDEDLEESPVPLNKDHPFPIDFELTP

EHEL_010945 MFTMDHTYYTDAFLDACLLGYTSIELTASSFTNIKDEEIEEVPIPLAKDHAFPIDFGLGP

ECU01_1065 MFNMDHTYYTDAFLDACLLGHTTIELTAKPFTNIKDEDIEEAPIPLARDQAFPIDFGLTP

EROM_010865 MFEMDHTYYTDAFLDACLLGYTSIELTAKPFTNVKDEDIEETPIPLTKDHVFPIDFGLTL

** ***************.. *****..***:***::** *:** .*: ***** *

EINT_010975 RNA----------

EHEL_010945 -------------

ECU01_1065 WERQDQASKRINN

EROM_010865 -------------

**Chromosome 2**

| Species | Positions |
| --- | --- |
| *E. cuniculi* | 29134..29307 |
| *E. hellem* | 20953..21126 |
| *E. intestinalis* | 17190..17369 |
| *E. romalae* | 15572..15745 |

>ECU02_0235

MVQRPLKNVKLHKKPTKYKRILKRAKSESELITKEINKGIIEKYRSLYEKKKSSSLK

>EHEL_020165

MVQRPLKNVKLHRKPTKYRKTLKKVKNENDLITKEINKGIIEKYKSMYEKKKNANIR

>EINT_020355

MVQRPLKNVKLHKKPTKYKRVLKKITSESDIITREINKGIVEKYMSLYKKKGKKNSDVR

>EROM_020335

MVQRPLKNVKLHKKPTKYKKTLKKVRNESDLITKEINKGIIEKYRSLYEKKRNTNLR

EINT_020355 MVQRPLKNVKLHKKPTKYKRVLKKITSESDIITREINKGIVEKYMSLYKKKGKKNSDVR

ECU02_0235 MVQRPLKNVKLHKKPTKYKRILKRAKSESELITKEINKGIIEKYRSLYEK--KKSSSLK

EHEL_020165 MVQRPLKNVKLHRKPTKYRKTLKKVKNENDLITKEINKGIIEKYKSMYEK--KKNANIR

EROM_020335 MVQRPLKNVKLHKKPTKYKKTLKKVRNESDLITKEINKGIIEKYRSLYEK--KRNTNLR

************.*****.. **. .*.::**.******:*** *:*:* *..:.:.

| Species | Positions |
| --- | --- |
| *E. cuniculi* | 49644..49817 |
| *E. hellem* | 41272..41445 |
| *E. intestinalis* | 37929..38117 |
| *E. romalae* | 35874..36047 |

>ECU02_0425

METKSRTSSAFRYIGVFIAILLCLSIVVDMMSFKRLSFLYEKSPKSELVELDRRTDK

>EHEL_020345

MELKSRTSASTFGYIGVFVAILLCLSIAVDMMSFKRLSFLEMPKSKPRHADGTPGER

>EINT_020355

MESKNRASASAFGFIGVFIAVLLCLSIVVDMMSFKRLSFLEVAESRHGIKMKEIMRNKEA

SE

>EROM_020335

MEQKSRTSASAFGYIGVFIAILLCLSIAVDMMSFKRLSFLEMLRSGPGHVNGRVDEG

EINT_020355 MESKNRASASAFGFIGVFIAVLLCLSIVVDMMSFKRLSFLEVAESRHGIKMKEIMRNKEA

ECU02_0425 METKSRTS-SAFRYIGVFIAILLCLSIVVDMMSFKRLSFLYEKSPKSELVELDRRTDK--

EHEL_020345 MELKSRTSASTFGYIGVFVAILLCLSIAVDMMSFKRLSFLEM--PKSKPRHADGTPGER-

EROM_020335 MEQKSRTSASAFGYIGVFIAILLCLSIAVDMMSFKRLSFLEM--LRSGPGHVNGRVDEG-

** *.*:* *:* :****:*:******.************ . : .:

EINT_020355 SE

ECU02_0425 --

EHEL_020345 --

EROM_020335 --

| Species | Positions |
| --- | --- |
| *E. cuniculi* | complement(104931..105110) |
| *E. hellem* | complement(96433..96636) |
| *E. intestinalis* | complement(93076..93333) |
| *E. romalae* | complement(91016..91231) |

>ECU02_0885

MTFKMVFITTVFLGSLAAFIMGLRRNNLLKLDLDAIKEATGITKSEIMVLGRRALEIFE

>EHEL_020805

MTMKMVFITTVFVGSLVAFIKGLHRDKMLKVDMSSIKEALVSTGNGIMALGRKTAETFTSKSEQDPT

>EINT_020835

MTLKMAFITTVFLASLTAFIMGLRRDKILKLDMETIKEAMEATGNEMAELGKKTMEVLVPSTSQNPEEPNESEADSNKESRKNLP

>EROM_020795

MTLKMVFITTVFLGSLAAFITGLRRDKMLKIDVGSIKEAFVSTGNGIVAFGRKTVEAFSPETGQGTDQDSK

EINT_020835 MTLKMAFITTVFLASLTAFIMGLRRDKILKLDMETIKEAMEATGNEMAELGKKTMEVLVP

ECU02_0885 MTFKMVFITTVFLGSLAAFIMGLRRNNLLKLDLDAIKEATGITKSEIMVLGRRALEIFE-

EHEL_020805 MTMKMVFITTVFVGSLVAFIKGLHRDKMLKVDMSSIKEALVSTGNGIMALGRKTAETFTS

EROM_020795 MTLKMVFITTVFLGSLAAFITGLRRDKMLKIDVGSIKEAFVSTGNGIVAFGRKTVEAFSP

**:**.******:.**.*** **.*:::**:*: :**** * . : :*..: * :

EINT_020835 STSQNPEEPNESEADSNKESRKNLP

ECU02_0885 -------------------------

EHEL_020805 KSEQDPT------------------

EROM_020795 ETGQGTDQDSK--------------

| Species | Positions |
| --- | --- |
| *E. cuniculi* | complement(182183..182353) |
| *E. hellem* | complement(173354..173524) |
| *E. intestinalis* | complement(170096..170266) |
| *E. romalae* | complement(168164..168334) |

>ECU02_1495

MEDGYKDVLEFANELKLHLTGDMIDQMYKLLDSGMSPRNLAQLLKEIRNELSGKIR

>EHEL_021435

MEDGYKDILEFANELKLHFNEDIIDYMYKLLDSGMSPQSLMLLLKEIRNELSGRVR

>EINT_021465

MEDGYKDILEFAKELKLHFGEDTIDQMYKLLDSGMSPKNLIQLLKEIRNELSGRIR

>EROM_021405

MEDVYKGILEFANELKLHFSEDIIDQMYKLLDSGMCPKSLILLLKEIRNELSGRIR

ECU02_1495 MEDGYKDVLEFANELKLHLTGDMIDQMYKLLDSGMSPRNLAQLLKEIRNELSGKIR

EINT_021465 MEDGYKDILEFAKELKLHFGEDTIDQMYKLLDSGMSPKNLIQLLKEIRNELSGRIR

EROM_021405 MEDVYKGILEFANELKLHFSEDIIDQMYKLLDSGMCPKSLILLLKEIRNELSGRIR

EHEL_021435 MEDGYKDILEFANELKLHFNEDIIDYMYKLLDSGMSPQSLMLLLKEIRNELSGRVR

*** **.:****:*****: * ** *********.*..* ***********.:*

**Chromosome 3**

| Species | Positions |
| --- | --- |
| *E. cuniculi* | 28954..29124 |
| *E. hellem* | 16806..16976 |
| *E. intestinalis* | 16357..16527 |
| *E. romalae* | 18019..18189 |

>ECU03_0255

MSTTKQEIQSQMEHADNLFEEMNRKMETLKGKLLNLSKMVDSINKKIEDMSMDKMA

>EHEL_030135

MSTTKQEIQNQMEHADNLFEEMNKKMEALKGKLLSLSKMVDGINKKIEDISTDSIA

>EINT_030145

MSTTKQEIQSQMEHADNLFVEMNKKMEALKGKLLSLSKMVDGINKKIEDISAESTG

>EROM_030155

MSTTKQEIQSQMEHADNLFEEMNKKMEALKGKLLSLSKMVDSINKKIEDISADPMA

EINT_030145 MSTTKQEIQSQMEHADNLFVEMNKKMEALKGKLLSLSKMVDGINKKIEDISAESTG

ECU03_0255 MSTTKQEIQSQMEHADNLFEEMNRKMETLKGKLLNLSKMVDSINKKIEDMSMDKMA

EHEL_030135 MSTTKQEIQNQMEHADNLFEEMNKKMEALKGKLLSLSKMVDGINKKIEDISTDSIA

EROM_030155 MSTTKQEIQSQMEHADNLFEEMNKKMEALKGKLLSLSKMVDSINKKIEDISADPMA

*********.********* ***.***:******.******.*******:* : .

**Chromosome 4**

| Species | Positions |
| --- | --- |
| *E. cuniculi* | complement(19114..19281) |
| *E. hellem* | complement(5757..5942) |
| *E. intestinalis* | complement(6889..7053) |
| *E. romalae* | complement(7608..7793) |

>ECU04_0123

MSLFPRKDTDGIRLENILKYLGILLVILLVTYSYQEMKKWVKRNVKGSSLAGGNK

>EHEL_040035

MSLFPKKEIEVIRPEGMLKYLGILFVVLLVTYSYQEIRKWVKRNMKNNLLVEEVNKEGPGF

>EINT_040045

MSLFPRREMEIMNPDGVVKYLGILFIVLLITYSYREMRKWVKRNIKSGGLVEGK

>EROM_040065

MSLFPKREIDTINPESMLKYLGILFIVLLVTYFYQETKKWVKRNIKNTILAEEINKEGLSY

ECU04_0123 MSLFPRKDTDGIRLENILKYLGILLVILLVTYSYQEMKKWVKRNVKGSSLAGGNK-----

EINT_040045 MSLFPRREMEIMNPDGVVKYLGILFIVLLITYSYREMRKWVKRNIKSGGLVEGK------

EROM_040065 MSLFPKREIDTINPESMLKYLGILFIVLLVTYFYQETKKWVKRNIKNTILAEEINKEGLS

EHEL_040035 MSLFPKKEIEVIRPEGMLKYLGILFVVLLVTYSYQEIRKWVKRNMKNNLLVEEVNKEGPG

*****..: : :. :.::******:::**:** *.* .******:*. *.

ECU04_0123 -

EINT_040045 -

EROM_040065 Y

EHEL_040035 F

| Species | Positions |
| --- | --- |
| *E. cuniculi* | complement(21451..21615) |
| *E. hellem* | complement(8083..8247) |
| *E. intestinalis* | complement(9220..9387) |
| *E. romalae* | complement(9966..10130) |

>ECU04_0152

MDRDEDLFARINEEEMEADVDQAVKNYEIPEIRVIEEFILSHDKLFQDIEKLNN

>EHEL_040072

MEEEEGLFDKINEAEMERDIELAIKGHRVPEIRPMEEFILSCDELFQNIEKFKH

>EINT_040082

MEEDESLFDKINEAEMEADIKLAMNNHKIPEIKPIEEFILSYNGIFQNLEELKKD

>EROM_040102

MGGEENLFGKLNESEMEKDIDLAIKSHKIPEIRSIEEFILSYDEVFQRIEKFKH

ECU04_0152 MDRDEDLFARINEEEMEADVDQAVKNYEIPEIRVIEEFILSHDKLFQDIEKLNN-

EINT_040082 MEEDESLFDKINEAEMEADIKLAMNNHKIPEIKPIEEFILSYNGIFQNLEELKKD

EHEL_040072 MEEEEGLFDKINEAEMERDIELAIKGHRVPEIRPMEEFILSCDELFQNIEKFKH-

EROM_040102 MGGEENLFGKLNESEMEKDIDLAIKSHKIPEIRSIEEFILSYDEVFQRIEKFKH-

* :*.** .:** *** *:. *::.: :***. :****** : :** :*::::

| Species | Positions |
| --- | --- |
| *E. cuniculi* | complement(205868..205954) |
| *E. hellem* | complement(189226..189312) |
| *E. intestinalis* | complement(193188..193274) |
| *E. romalae* | complement(191322..191408) |

>ECU04_1622

MALLEKTSRKILKFMILVLFSYIVLSIN

>EHEL_041595

MAPFEKASRKIIKFMILVLFSYIVLSIN

>EINT_041635

MNSFEKKTRNIIKFLILVLFSYIVLSIN

>EROM_041652

MASFEKASRRIIKFMILVLFSYIVLSIN

ECU04_1622 MALLEKTSRKILKFMILVLFSYIVLSIN

EHEL_041595 MAPFEKASRKIIKFMILVLFSYIVLSIN

EROM_041652 MASFEKASRRIIKFMILVLFSYIVLSIN

EINT_041635 MNSFEKKTRNIIKFLILVLFSYIVLSIN

* :** :*.*:**:*************

| Species | Positions |
| --- | --- |
| *E. cuniculi* | 207673..207840 |
| *E. hellem* | - |
| *E. intestinalis* | - |
| *E. romalae* | 194418..194585 |

>ECU04_1635

MNSGKYVESIEKIELLLKSLRSVLNGIKNGAVNQKDIDNIKESLFAIKANARSCL

>EROM_041665

MDSEKYKKSIESIELLLKSLEGVLDGIKRRAVDPKDINNIRESLLTIKTNARECL

ECU04_1635 MNSGKYVESIEKIELLLKSLRSVLNGIKNGAVNQKDIDNIKESLFAIKANARSCL

EROM_041665 MDSEKYKKSIESIELLLKSLEGVLDGIKRRAVDPKDINNIRESLLTIKTNARECL

*:* ** :***.******** .**:***. **: ***:**.***::**:***.**

**Chromosome 5**

| Species | Positions |
| --- | --- |
| *E. cuniculi* | complement(17320..17535) |
| *E. hellem* | complement(20638..20844) |
| *E. intestinalis* | complement(14231..14431) |
| *E. romalae* | complement(9320..9517) |

>ECU05_0087

MRMVHCILSTSLCITGILTDVNSQSLLKAEELERQLLNAEDILMMEHLRRQGFIIYPSKASGRALNDLSLH

>EHEL_050137

MRVTHCILFINLCIAQTLTNTDNKNLLEAEALEKKLLHAEDILMMEHLKEQGFTVYPNKTPGSTNNTY

>EINT_050075

MRIVRYILSINLCMMNVLANTRSKSLLEGERLEKKLLNEEDILMMEHLKDQGFIIIYPSKANNSFQ

>EROM_050055

MRVIYYILSINLCIARTLTNANSKSLLEAEGLEKRLLHAEDILMMEHLKDQGFIVYPSRTLSSAE

ECU05_0087 MRMVHCILSTSLCITGILTDVNSQSLLKAEELERQLLNAEDILMMEHLRRQGF-IIYPSK

EINT_050075 MRIVRYILSINLCMMNVLANTRSKSLLEGERLEKKLLNEEDILMMEHLKDQGFIIIYPSK

EHEL_050137 MRVTHCILFINLCIAQTLTNTDNKNLLEAEALEKKLLHAEDILMMEHLKEQGF-TVYPNK

EROM_050055 MRVIYYILSINLCIARTLTNANSKSLLEAEGLEKRLLHAEDILMMEHLKDQGF-IVYPSR

**: ** .**: *::. .:.**:.* **..**: *********. *** :**..

ECU05_0087 ASGRALNDLSLH

EINT_050075 ANNSFQ------

EHEL_050137 TPGSTNNTY---

EROM_050055 TLSSAE------

: .

| Species | Positions |
| --- | --- |
| *E. cuniculi* | complement(20245..20442) |
| *E. hellem* | complement(23557..23751) |
| *E. intestinalis* | complement(17145..17342) |
| *E. romalae* | complement(12222..12437) |

>ECU05_0115

MKRKMLHLCLGIGMIGARRYKLGYGLRGPLHSLESRLYKGWLSNYQPQLGFFSDSSSGQCDSSSD

>EHEL_050165

MKRKIQLLCLFLKIAVTRRHRLGYGLRGSHLSLESKLYNSWLADHQALTDSSGNTNNDLDFRAY

>EINT_050105

MKRKMQLVYLTLRMVVARQNSLGYGLRESQNSLEARLYKNWIPTHKSWSSFLSNNIEESCDSSSD

>EROM_050085

MKRKMQFLYLFLEIIMARQYRLGYGLRGSHLSLKSKLHGSWLTDHQPSHDSSLNNVQDLNNSFSSSSDSTQ

EINT_050105 MKRKMQLVYLTLRMVVARQNSLGYGLRESQNSLEARLYKNWIPTHKSWSSFLSNNIEESC

ECU05_0115 MKRKMLHLCLGIGMIGARRYKLGYGLRGPLHSLESRLYKGWLSNYQPQLGFFSDSSSGQC

EHEL_050165 MKRKIQLLCLFLKIAVTRRHRLGYGLRGSHLSLESKLYNSWLADHQALTDSSGNTNNDLD

EROM_050085 MKRKMQFLYLFLEIIMARQYRLGYGLRGSHLSLKSKLHGSWLTDHQPSHDSSLNNVQDLN

****: : * : : :*. ****** . **::.*: .*:. ::. . :. .

EINT_050105 DSSSD------

ECU05_0115 DSSSD------

EHEL_050165 FRAY-------

EROM_050085 NSFSSSSDSTQ

| Species | Positions |
| --- | --- |
| *E. cuniculi* | 157270..157425 |
| *E. hellem* | 160953..161111 |
| *E. intestinalis* | 154308..154457 |
| *E. romalae* | 149671..149829 |

>ECU05_1185

MNNRKKFEEIKQQLESFLTPRHLRNEFPHEKGWVVPESMSHESELRKDGIQ

>EHEL_051295

MNNRKKFEEIKQQLESFLTPRHLRNEFPRKKGWLVPESTAHETELRKESKIQ

>EINT_051235

MNNRKKFEEIKQQLESFLTPKHLRNEFPQEKGWMMPENSPYKNEYRKEG

>EROM_051225

MNNKKKFEEIKKQLESFLTPRHLRNEFPREKRWVVPESTAHESELRRENKIQ

EINT_051235 MNNRKKFEEIKQQLESFLTPKHLRNEFPQEKGWMMPENSPYKNEYRKEG---

ECU05_1185 MNNRKKFEEIKQQLESFLTPRHLRNEFPHEKGWVVPESMSHESELRKDG-IQ

EROM_051225 MNNKKKFEEIKKQLESFLTPRHLRNEFPREKRWVVPESTAHESELRRENKIQ

EHEL_051295 MNNRKKFEEIKQQLESFLTPRHLRNEFPRKKGWLVPESTAHETELRKESKIQ

***.*******:********.*******.:* *::**. .::.* *.:.

| Species | Positions |
| --- | --- |
| *E. cuniculi* | 168182..168310 |
| *E. hellem* | 172865..172978 |
| *E. intestinalis* | 166265..166378 |
| *E. romalae* | 161617..161730 |

>ECU05_1275

MYFKYDKDMMEKIIRKLTADSSGNRRTEDQLKAGEEASVNEH

>EHEL_051395

MYFEYDKNIMRSIVKKLMDDTSKDKENEEQAKDGEEI

>EINT_051335

MYFEYDKAIMQKIIKKLKEESDEDQGIEDHNKGNGEE

>EROM_051335

MYFEYDKNVMRSIVKRLMEDSSKDKDAEDQTKDSEDT

ECU05_1275 MYFKYDKDMMEKIIRKLTADSSGNRRTEDQLKAGEEASVNEH

EHEL_051395 MYFEYDKNIMRSIVKKLMDDTSKDKENEEQAKDGEEI-----

EROM_051335 MYFEYDKNVMRSIVKRLMEDSSKDKDAEDQTKDSEDT-----

EINT_051335 MYFEYDKAIMQKIIKKLKEESDEDQGIEDHNKGNGEE-----

***:*** :* .*:..* ::. :. *:: * . :

**Chromosome 6**

| Species | Positions |
| --- | --- |
| *E. cuniculi* | 40665..40766 |
| *E. hellem* | 30724..30822 |
| *E. intestinalis* | 27126..27230 |
| *E. romalae* | 26393..26491 |

>ECU06_0285

MFYTALFVNVLIYHSLWRNLLRHLFNKTSYSIG

>EHEL_060205

MFYTALFINVLIYHGLWRNLLRHLFNKSYSLG

>EINT_060185

MFYTALFVNIIIYHGLWRNLLKHLFIKTSSFPIG

>EROM_060195

MFYTALFVNVLIYHGLWRNLLRHLFNKSYSLS

EINT_060185 MFYTALFVNIIIYHGLWRNLLKHLFIKTSSFPIG

ECU06_0285 MFYTALFVNVLIYHSLWRNLLRHLFNKT-SYSIG

EHEL_060205 MFYTALFINVLIYHGLWRNLLRHLFNK--SYSLG

EROM_060195 MFYTALFVNVLIYHGLWRNLLRHLFNK--SYSLS

*******:*::***.******.*** * *:.:.

**Chromosome 7**

| Species | Positions |
| --- | --- |
| *E. cuniculi* | 111237..111362 |
| *E. hellem* | 106349..106468 |
| *E. intestinalis* | 102189..102314 |
| *E. romalae* | 102324..102443 |

>ECU07_0862

MAGLSQEKKRMYLEEIEELEKILENEEDKLRSIRRPQSFGQ

>EHEL_070832

MTELSQEKREMYLKEIEELEKTLKEEEDELKKITKTEDF

>EINT_070802

MAELSEEKRRMYLEQIEELEKTLEKEKDSLRKTIESQDSKQ

>EROM_070812

MAELSQEKRRMYLKEIEELERILEDEEDELKRIVSPEGS

EINT_070802 MAELSEEKRRMYLEQIEELEKTLEKEKDSLRKTIESQDSKQ

ECU07_0862 MAGLSQEKKRMYLEEIEELEKILENEEDKLRSIRRPQSFGQ

EHEL_070832 MTELSQEKREMYLKEIEELEKTLKEEEDELKKITKTEDF--

EROM_070812 MAELSQEKRRMYLKEIEELERILEDEEDELKRIVSPEGS--

*: **:**. ***::*****. *:.*:*.*. .:.

| Species | Positions |
| --- | --- |
| *E. cuniculi* | complement(160835..161089) |
| *E. hellem* | complement(154656..154910) |
| *E. intestinalis* | complement(151750..152004) |
| *E. romalae* | complement(150781..151035) |

>ECU07_1385

MVEIKIKKIYPVYSWKWDIESDICGICQQGFDQMCTKCKHPMECKPCVGKCKHTFHSHCIALWLQQRKVCPMCRVFWVCHRAFE

>EHEL_071365

MVEIKIKKVYPVYNWKWDIESDICGICQQSFDQMCIKCKHPMECKPCIGKCKHTFHSHCIALWLQQRKVCPMCRVFWVCQRVFE

>EINT_071345

MVEIKIKKVYPVYNWKWDIESDICGICQQSFDQMCIKCKHPIECKPCIGKCKHTFHSHCIALWLQQRKICPMCRVYWICHRMFE

>EROM_071325

MVEIKIKKVYPVYNWRWDIESDICGICQQSFDQMCIKCKHPIECKPCIGKCKHTFHSHCIALWLQQRKVCPMCRVFWVCHRVFE

ECU07_1385 MVEIKIKKIYPVYSWKWDIESDICGICQQGFDQMCTKCKHPMECKPCVGKCKHTFHSHCI

EINT_071345 MVEIKIKKVYPVYNWKWDIESDICGICQQSFDQMCIKCKHPIECKPCIGKCKHTFHSHCI

EHEL_071365 MVEIKIKKVYPVYNWKWDIESDICGICQQSFDQMCIKCKHPMECKPCIGKCKHTFHSHCI

EROM_071325 MVEIKIKKVYPVYNWRWDIESDICGICQQSFDQMCIKCKHPIECKPCIGKCKHTFHSHCI

********:****.*.*************.***** *****:*****:************

ECU07_1385 ALWLQQRKVCPMCRVFWVCHRAFE

EINT_071345 ALWLQQRKICPMCRVYWICHRMFE

EHEL_071365 ALWLQQRKVCPMCRVFWVCQRVFE

EROM_071325 ALWLQQRKVCPMCRVFWVCHRVFE

********:******:*:*:* **

| Species | Positions |
| --- | --- |
| *E. cuniculi* | complement(195045..195254) |
| *E. hellem* | complement(188682..188894) |
| *E. intestinalis* | complement(185658..185876) |
| *E. romalae* | complement(184630..184842) |

>ECU07_1645

MKREGENGEGKSKDLKDKRRKKTEEEIERIRAHRIAERRLYMQRTKKNQPVTSNRISLLLKQIEKLPKE

>EHEL_071625

MKAGNKEEKIKRIQDRRSGKSEEEIERIRSHRSARIKLYMQRTRRNQPVTRNRISLLL KRIEEFQGRNFN

>EINT_071615

MKYRKEDGKKQDKSIRKRENRKTEEEIERIRAQKAVQRRLYMQKTRRNQPVTGNRINLLLKQIEKFQRNSSG

>EROM_071565

MKVGNGKKKEEKVQDRRGIKAEEEIKRIRDRRNAKNKLYMQRTRKNQPVTGNRISLLLKQIEELQGSNIN

EINT_071615 MKYRKEDGKKQDKSIRKRENRKTEEEIERIRAQKAVQRRLYMQKTRRNQPVTGNRINLLL

EHEL_071625 --MKAGNKEEKIKRIQDRRSGKSEEEIERIRSHRSARIKLYMQRTRRNQPVTRNRISLLL

EROM_071565 --MKVGNGKKKEEKVQDRRGIKAEEEIKRIRDRRNAKNKLYMQRTRKNQPVTGNRISLLL

ECU07_1645 MKREGENGEGKSKDLKDKRRKKTEEEIERIRAHRIAERRLYMQRTKKNQPVTSNRISLLL

: : : : :... *:****:*** .. . .****.*..***** ***.***

EINT_071615 KQIEKFQRNSSG

EHEL_071625 KRIEEFQGRNFN

EROM_071565 KQIEELQGSNIN

ECU07_1645 KQIEKLPKE---

*.**::

| Species | Positions |
| --- | --- |
| *E. cuniculi* | 211391..211618 |
| *E. hellem* | 205019..205210 |
| *E. intestinalis* | 201864..202001 |
| *E. romalae* | 200970..201224 |

>ECU07_1775

MRVLLVILMLATAVVLIVTMTVILENINRKPKNRFPDSSDDSSGGLGKGTGSGDSWNYSPDSSTSLTDLEDGMQK

>EHEL_071755

MRVLLVILILAAAIVLIVTMTVVLENIDRKSEDESEDSFDGNYPCHSEKESGNSENHLPESIQ

>EINT_071745

MRVLLVILMLATAIVLTVTLTIILENMDRKGDDDPSDSFDGNSSG

>EROM_071695

MRFLLVILMLAAAVVLIVTMTIVLENINRKPEDEFSDSFDDSPFDEEEESGNSKNYLFESSSQLEEVEIKDEFVNSVLITELMH

ECU07_1775 MRVLLVILMLATAVVLIVTMTVILENINRKPKNRFPDSSDDSSGGLGKGTGSGDSWNYSP

EROM_071695 MRFLLVILMLAAAVVLIVTMTIVLENINRKPEDEFSDSFDDS--PFDEEEESGNSKNYLF

EHEL_071755 MRVLLVILILAAAIVLIVTMTVVLENIDRKSEDESEDSFDGNY-PCHSEKESGNSENHLP

EINT_071745 MRVLLVILMLATAIVLTVTLTIILENMDRKGDDDPSDSFDGN--------SSG-------

**.*****:**:*:** **:*::***::** .: ** *.. **

ECU07_1775 DSSTSLTDLEDGMQK-----------

EROM_071695 ESSSQLEEVEIKDEFVNSVLITELMH

EHEL_071755 ESIQ----------------------

EINT_071745 --------------------------

**Chromosome 8**

| Species | Positions |
| --- | --- |
| *E. cuniculi* | 162830..163012 |
| *E. hellem* | 144958..145146 |
| *E. intestinalis* | 144813..144995 |
| *E. romalae* | 145368..145550 |

>ECU08_1445

MGDIFSPIQVQIEKIEDEVERLYRMVRRMVRQHAKISRFLMLQERVLTRRVRDQQSGEEL

>EHEL_081425

MGDIFSPIQVQIERIEKQIEKLCTTVRRMVSQNAKISRFLVSQERILARRSHGQANGGGTEC

>EINT_081425

MGDIFSPIQVQLERIERQMEKLCTGIGRMVEQHSKIGRFLIAQEKILTRRTQTLQNGEAL

>EROM_081445

MGDVFSPIQAQIERIEREIERLCTTVGRMIRQNIKISRFLASQERILTRRPHRQQDKDDL

EINT_081425 MGDIFSPIQVQLERIERQMEKLCTGIGRMVEQHSKIGRFLIAQEKILTRRTQTLQNGEAL

ECU08_1445 MGDIFSPIQVQIEKIEDEVERLYRMVRRMVRQHAKISRFLMLQERVLTRRVRDQQSGEEL

EROM_081445 MGDVFSPIQAQIERIEREIERLCTTVGRMIRQNIKISRFLASQERILTRRPHRQQDKDDL

EHEL_081425 MGDIFSPIQVQIERIEKQIEKLCTTVRRMVSQNAKISRFLVSQERILARRSHGQANGGGT

***:*****.*:*.** ::*.* : **: *: **.*** **.:*:** . .

EINT_081425 --

ECU08_1445 --

EROM_081445 --

EHEL_081425 EC

| Species | Positions |
| --- | --- |
| *E. cuniculi* | complement(172228..172386) |
| *E. hellem* | complement(154285..154443) |
| *E. intestinalis* | complement(154143..154301) |
| *E. romalae* | complement(154748..154906) |

>ECU08_1555

MFYRLEDGARVYTLDENGTISAHPAKFTVEDRFSKERIAIKRRFAIPPFNNL

>EHEL_081525

MFYRLKDGERIYTLDEEETFCAHPAKFTIEDKFSKERITIKKRFNIPPFNDL

>EINT_081525

MFYRLKDGERVYTLSEEDSFSAHPAKFTIEDKFSKERITIKKRFNIPPFNDL

>EROM_081555

MFYRLKDGERIYTLDEEGTFSAHPAKFTIEDKFSKERITIKKRFNIPPFNGL

ECU08_1555 MFYRLEDGARVYTLDENGTISAHPAKFTVEDRFSKERIAIKRRFAIPPFNNL

EINT_081525 MFYRLKDGERVYTLSEEDSFSAHPAKFTIEDKFSKERITIKKRFNIPPFNDL

EHEL_081525 MFYRLKDGERIYTLDEEETFCAHPAKFTIEDKFSKERITIKKRFNIPPFNDL

EROM_081555 MFYRLKDGERIYTLDEEGTFSAHPAKFTIEDKFSKERITIKKRFNIPPFNGL

*****:** *:***.*: ::.*******:**.******:**.** *****.*

**Chromosome 9**

| Species | Positions |
| --- | --- |
| *E. cuniculi* | 62066..62194 |
| *E. hellem* | 59234..59362 |
| *E. intestinalis* | 60676..60804 |
| *E. romalae* | 59123..59251 |

>ECU09_0465

MEEHSEKELEKRLPSIRDLMKKSDDFWCKDDDEPVNTNRFAM

>EHEL_090465

MERHPEKEDRKELFDILGLIKRSDEFWLEDDDRVSLTNRFVV

>EINT_090475

MEECFEKENKKGWISIQDSMKRSNDFWLKDDDATSYLNRFVV

>EROM_090475

MEGYSEKESRKELFTIQDLMKRSDEFWSKDDDHLGYTNRFVV

ECU09_0465 MEEHSEKELEKRLPSIRDLMKKSDDFWCKDDDEPVNTNRFAM

EINT_090475 MEECFEKENKKGWISIQDSMKRSNDFWLKDDDATSYLNRFVV

EHEL_090465 MERHPEKEDRKELFDILGLIKRSDEFWLEDDDRVSLTNRFVV

EROM_090475 MEGYSEKESRKELFTIQDLMKRSDEFWSKDDDHLGYTNRFVV

** *** * * . :*.*::** :*** ***.:

| Species | Positions |
| --- | --- |
| *E. cuniculi* | 143995..144072 |
| *E. hellem* | complement(153284..153361) |
| *E. intestinalis* | complement(156971..157063) |
| *E. romalae* | 9184..9261 |

>ECU09_1255

MHNSQKSLEEILDRYNEVLQVSASI

>EHEL_091435

MNNFEQSLEEILDKYNDILQVSGFI

>EINT_091465

MDSFEKSLEEVLDRYNDILQVSEFPLTLGI

>EROM_090625

MYNFEQSLEEVLNEYNEILQVSESI

ECU09_1255 MHNSQKSLEEILDRYNEVLQVSAS-----I

EROM_090625 MYNFEQSLEEVLNEYNEILQVSES-----I

EHEL_091435 MNNFEQSLEEILDKYNDILQVSGF-----I

EINT_091465 MDSFEKSLEEVLDRYNDILQVSEFPLTLGI

* . ::****:*: **::**** *

| Species | Positions |
| --- | --- |
| *E. cuniculi* | complement(193409..193558) |
| *E. hellem* | complement(14531..14680) |
| *E. intestinalis* | complement(180357..180506) |
| *E. romalae* | complement(14985..15134) |

>ECU09_1665

MDYSSKLKVIRETSVSPEEAKRMIEEFVLSSDVLEGIQVLQLEEFLNNV

>EHEL_091675

MDYSSKVEVIEEASLVTEEAKEMVRKFILNNDSLEGIQILQLEEFLNNV

>EINT_091675

MDYNSKLEIAREVSMNPEDAKEIIREFMMSNDSLEGIQILQLEEFLNHV

>EROM_091655

MDYNSKIKVVEERSLTPEKAKEMLREFLLDNDDLEGIQVLQLEEFLNNV

ECU09_1665 MDYSSKLKVIRETSVSPEEAKRMIEEFVLSSDVLEGIQVLQLEEFLNNV

EROM_091655 MDYNSKIKVVEERSLTPEKAKEMLREFLLDNDDLEGIQVLQLEEFLNNV

EHEL_091675 MDYSSKVEVIEEASLVTEEAKEMVRKFILNNDSLEGIQILQLEEFLNNV

EINT_091675 MDYNSKLEIAREVSMNPEDAKEIIREFMMSNDSLEGIQILQLEEFLNHV

***.**::: * *: .*.** :: :*::..* *****:********:*

| Species | Positions |
| --- | --- |
| *E. cuniculi* | 208093..208224 |
| *E. hellem* | 29180..29314 |
| *E. intestinalis* | 194983..195114 |
| *E. romalae* | 29673..29807 |

>ECU09_1755

MGNKRRQSFEQEFIDSFVSDEVINWRFDPILLDDILRTRLDQE

>EHEL_091775

MANKKRQSFEQEFIDSFVSDEVIHWKFDPILLDDILKDRIGQEE

>EINT_091775

MGNKRRPSFEQEFIDSFVSDEVINWKFDPILLDGELKARLSQE

>EROM_091755

MANKKRPSFEQEFIDSFVSDEVINWRFDMILLDDTLKARLGQEE

EHEL_091775 MANKKRQSFEQEFIDSFVSDEVIHWKFDPILLDDILKDRIGQEE

EROM_091755 MANKKRPSFEQEFIDSFVSDEVINWRFDMILLDDTLKARLGQEE

ECU09_1755 MGNKRRQSFEQEFIDSFVSDEVINWRFDPILLDDILRTRLDQE-

EINT_091775 MGNKRRPSFEQEFIDSFVSDEVINWKFDPILLDGELKARLSQE-

*.**.* ****************:*.** ****. *. *:.**

**Chromosome 10**

| Species | Positions |
| --- | --- |
| *E. cuniculi* | 78695..78901 |
| *E. hellem* | 69349..69555 |
| *E. intestinalis* | 66120..66326 |
| *E. romalae* | 57423..57629 |

>ECU10_0635

MLDKGSEEKLLTDYEPKRCRKMPRERKSTQASCQESRRRGSLFRVDLAKVIAKKEHFVFSGFLDKFIT

>EHEL_100635

MLDKGSEEKYLTNYEPRTYPKRARKETCEQISLIAPRRRGSLFRIDLPKVMEEREYFTFSRFLNRFIR

>EINT_100575

MLDKGSKEKLLTSYEPRTYLRRPRKKEDEQMSQTPLRRHGSLFRVDLSRVIKEKEHFVFSRFLNRFIR

>EROM_100505

MLDKGSEEKLLTDYEPRTYRERSKERRFKDVALKMPRRRGSLFRVDLSKVVEEKKYFIFSTFLNRFIK

ECU10_0635 MLDKGSEEKLLTDYEPKRCRKMPRERKSTQASCQESRRRGSLFRVDLAKVIAKKEHFVFS

EROM_100505 MLDKGSEEKLLTDYEPRTYRERSKERRFKDVALKMPRRRGSLFRVDLSKVVEEKKYFIFS

EINT_100575 MLDKGSKEKLLTSYEPRTYLRRPRKKEDEQMSQTPLRRHGSLFRVDLSRVIKEKEHFVFS

EHEL_100635 MLDKGSEEKYLTNYEPRTYPKRARKETCEQISLIAPRRRGSLFRIDLPKVMEEREYFTFS

******:** **.***. ..: : : **.*****:**..*: :.::* **

ECU10_0635 GFLDKFIT

EROM_100505 TFLNRFIK

EINT_100575 RFLNRFIR

EHEL_100635 RFLNRFIR

**:.**

**Chromosome 11**

| Species | Positions |
| --- | --- |
| *E. cuniculi* | complement(23251..23418) |
| *E. hellem* | complement(5632..5793) |
| *E. intestinalis* | complement(6894..7055) |
| *E. romalae* | complement(4199..4366) |

>ECU11_0185

MFEKLALAAEKVKNSRKGCLNITSEEVESLVCPIRFDSDECIEIMCRGSRAFDYK

>EHEL_110065

MIKKLVLATEKAKNSRKGNLKITSEEIESLICPIRFNSDECVEIMCKNDKMFN

>EINT_110055

MFEKLSLAMEKAKNSRKGCLNVTSEEIESLVCPIKFNSDECIEIMCRNCKEFD

>EROM_110055

MFEKLVLATEKAKNSRKGNLNTTSEEIESLVCPMKFNSDECIEIMCKNPKVLNYK

EHEL_110065 MIKKLVLATEKAKNSRKGNLKITSEEIESLICPIRFNSDECVEIMCKNDKMFN--

ECU11_0185 MFEKLALAAEKVKNSRKGCLNITSEEVESLVCPIRFDSDECIEIMCRGSRAFDYK

EINT_110055 MFEKLSLAMEKAKNSRKGCLNVTSEEIESLVCPIKFNSDECIEIMCRNCKEFD--

EROM_110055 MFEKLVLATEKAKNSRKGNLNTTSEEIESLVCPMKFNSDECIEIMCKNPKVLNYK

*::** ** **.****** *: ****:***:**:.*:****:****.. . ::

| Species | Positions |
| --- | --- |
| *E. cuniculi* | 75069..75257 |
| *E. hellem* | 48637..48819 |
| *E. intestinalis* | 49726..49920 |
| *E. romalae* | 47143..47325 |

>ECU11_0525

MGVRESFINVRRSCGPSEDPDNDDHEEEYYKWRIRDLKRKIRDIKKEIAKAKNIGDTDPRIY

>EHEL_110395

MNIRESFINARRACASPTDSDRDSYEEEYYKWKIRDLKRKIRDVRKEVAKARNIRGLRRF

>EINT_110375

MNVRESFISTRKTNVSREDSEKDCYEEEYYKWRIRDLKRKIRDIRKEIIRAKNIGTSGNDPKIY

>EROM_110385

MNIRESFINARRTEVSPANLDKDNYEEEYYKWKIRDLKRKIRDVRKEVARVRNIRGLGKS

EHEL_110395 MNIRESFINARRACASPTDSDRDSYEEEYYKWKIRDLKRKIRDVRKEVAKARNIR--G--

EROM_110385 MNIRESFINARRTEVSPANLDKDNYEEEYYKWKIRDLKRKIRDVRKEVARVRNIR--G--

EINT_110375 MNVRESFISTRKTNVSREDSEKDCYEEEYYKWRIRDLKRKIRDIRKEIIRAKNIGTSGND

ECU11_0525 MGVRESFINVRRSCGPSEDPDNDDHEEEYYKWRIRDLKRKIRDIKKEIAKAKNIG--DTD

*.:*****..*.: . : :.* :*******.**********:.**: ...** .

EHEL_110395 LRRF

EROM_110385 LGKS

EINT_110375 PKIY

ECU11_0525 PRIY

| Species | Positions |
| --- | --- |
| *E. cuniculi* | complement(80609..80764) |
| *E. hellem* | complement(54148..54324) |
| *E. intestinalis* | complement(55245..55415) |
| *E. romalae* | complement(52669..52839) |

>ECU11_0575

MKEKIERAYNRFVRSLDGGIRSRGGASIVENVEELENLLEELIFQLEDVEL

>EHEL_110445

MKEKIERAYNRLIRSLDSGIRLGGTSISESIEELERVLEDMIIQLEDDGFEVFKTSEV

>EINT_110425

MKENIEKAYNRLIRGLDSGIRLDGASISESIESLEAILEELVVQLEDPGFDFLEDK

>EROM_110435

MKEKIERAYNRLIRSLDSGVRTGGASISESVEELERILEDIMIHLEDEDFNIFKTQ

EINT_110425 MKENIEKAYNRLIRGLDSGIRL-DGASISESIESLEAILEELVVQLEDPGFDFLEDK--

ECU11_0575 MKEKIERAYNRFVRSLDGGIRSRGGASIVENVEELENLLEELIFQLEDVEL--------

EHEL_110445 MKEKIERAYNRLIRSLDSGIRL-GGTSISESIEELERVLEDMIIQLEDDGFEVFKTSEV

EROM_110435 MKEKIERAYNRLIRSLDSGVRT-GGASISESVEELERILEDIMIHLEDEDFNIFKTQ--

***:**.****::*.**.*:* .*:** *.:*.** :**:::.:*** :

| Species | Positions |
| --- | --- |
| *E. cuniculi* | 148601..148855 |
| *E. hellem* | 122021..122272 |
| *E. intestinalis* | 123149..123403 |
| *E. romalae* | 120614..120868 |

>ECU11_1175

MGPGEAQNNCPNLPPGMDIEDLIKSADNFFRGRPSSNPMVSSKKKVLRFAKDNGVDMNKSLIKAEEFPRSAYADELERFPVLRD

>EHEL_111055

MGLGKEENNCQRLSDLDMEDLARKIDTFFSNRKTTNLMASSKKKVLQFAKSRGVNMNKSLIEVEEFPRNAYADELERFPVLKD

>EINT_111055

MGFDKTENNSWSFSVSADVRTMNQKADAFFRARQNSNPVVSTKKKILQFAKDSQVDMNKSLIEIEEFPRNAYADELERFPVLKD

>EROM_111055

MGPSKNKNNCQACPFTMDTEDLARKVENFFSNRKASNPILSSKKKVFQFARSKGVDMNKSLIEVEEFPRHAYADELERFPILRD

EINT_111055 MGFDKTENNSWSFSVSADVRTMNQKADAFFRARQNSNPVVSTKKKILQFAKDSQVDMNKS

ECU11_1175 MGPGEAQNNCPNLPPGMDIEDLIKSADNFFRGRPSSNPMVSSKKKVLRFAKDNGVDMNKS

EHEL_111055 MGLGKEENNCQRLS-DLDMEDLARKIDTFFSNRKTTNLMASSKKKVLQFAKSRGVNMNKS

EROM_111055 MGPSKNKNNCQACPFTMDTEDLARKVENFFSNRKASNPILSSKKKVFQFARSKGVDMNKS

** .: :**. . * : .. : ** * :* : *:***::.**.. *:****

EINT_111055 LIEIEEFPRNAYADELERFPVLKD

ECU11_1175 LIKAEEFPRSAYADELERFPVLRD

EHEL_111055 LIEVEEFPRNAYADELERFPVLKD

EROM_111055 LIEVEEFPRHAYADELERFPILRD

**: ***** **********:*.*

| Species | Positions |
| --- | --- |
| *E. cuniculi* | complement(154202..154354) |
| *E. hellem* | complement(127566..127739) |
| *E. intestinalis* | complement(128704..128877) |
| *E. romalae* | complement(126208..126360) |

>ECU11_1205

MSLKEKFDLLRASIVECDQALRRLEQKSKILTQHVETSVLMARARENKDA

>EHEL_111085

MALQEKLRVLRNSIAECDQALRRLEQKSKILTQHVETSVLMAKSREGKEECLFQNEI

>EINT_111085

MSLKEKFDVLRNSIAECDQALRKLEQRSKILTQHVETSILMAKSRETKKQGTGETPS

>EROM_111085

MVLEEKIKLLRNSIVECDQALRKLEQRSKMLTQHVETSILMARSEESKEA

EROM_111085 MVLEEKIKLLRNSIVECDQALRKLEQRSKMLTQHVETSILMARSEESKEA-------

EHEL_111085 MALQEKLRVLRNSIAECDQALRRLEQKSKILTQHVETSVLMAKSREGKEECLFQNEI

ECU11_1205 MSLKEKFDLLRASIVECDQALRRLEQKSKILTQHVETSVLMARARENKDA-------

EINT_111085 MSLKEKFDVLRNSIAECDQALRKLEQRSKILTQHVETSILMAKSRETKKQGTGETPS

* *:**: :** **.*******.***.**:********:***.: * *.

| Species | Positions |
| --- | --- |
| *E. cuniculi* | complement(214696..214866) |
| *E. hellem* | complement(187863..188030) |
| *E. intestinalis* | complement(188885..189091) |
| *E. romalae* | complement(186541..186711) |

>ECU11_1725

MLTRITLDKMNERYAGAYYSPKPSELPAPEAVATIPLTFRINKKSLRCSNYDTNNS

>EHEL_111615

MLARITLDKMNERYAGVYYSPRPSELPTPDVVDIIPLAHRLSKEGIRRICHDTDK

>EINT_111615

MLTRITLDKMNERYAGVYYSPRPSELPSLETVRIVRLSHKQKKKILTAPAMKSTIIGCAL PSPSGKDN

>EROM_111615

MLTRITLDKMNERYAGVYYSPRPSELPAPEVVGIIPVAHKLSKEDIHRMCHNNGNK

EINT_111615 MLTRITLDKMNERYAGVYYSPRPSELPSLETVRIVRLSHKQKKKILTAPAMKSTIIGCAL

ECU11_1725 MLTRITLDKMNERYAGAYYSPKPSELPAPEAVATIPLTFRINKKSLRCSNYDT-------

EHEL_111615 MLARITLDKMNERYAGVYYSPRPSELPTPDVVDIIPLAHRLSKEGIRRICHDT-------

EROM_111615 MLTRITLDKMNERYAGVYYSPRPSELPAPEVVGIIPVAHKLSKEDIHRMCHNN-------

**:*************.****.*****: :.* : ::.. .*: : ..

EINT_111615 PSPSGKDN

ECU11_1725 -----NNS

EHEL_111615 ------DK

EROM_111615 -----GNK

:.
